# Precision medicine: an approach to R&D for delivering superior medicines to patients

**DOI:** 10.1186/2001-1326-1-7

**Published:** 2012-05-30

**Authors:** Mikael Dolsten, Morten Søgaard

**Affiliations:** 1 Pfizer World-wide R&D, 235 East 42nd Street, New York, NY, 10017, USA

## Abstract

Pharmaceutical R&D productivity has declined over the last decade despite increasing investments. Recent trends, however, indicate a potential reversal of this trend fueled by a wave of new biologics, vaccines and highly selective NCEs directed against targets validated by human genetics, focus on new disease areas including orphan and genetic diseases and more precise tailoring of medicines to their target populations.

## Precision medicine

Precision Medicine [1,2] is an approach to discovering and developing medicines and vaccines that deliver superior outcomes for patients, by integrating clinical and molecular information to understand the biological basis of disease. This approach leads to better selection of disease targets and identification of patient populations that experience better clinical outcomes. Ultimately, the potential of Precision Medicine is that it will yield treatments that deliver clinically significant treatment effects, with favorable safety profiles. When appropriate, these new treatments are focused on a particular sub- group of patients with certain genotypic and/or phenotypic characteristics that make them more likely to benefit or less likely to experience side effects. A related concept, “Personalized Medicine”, has been defined by President’s Council of Advisors on Science and Technology (PCAST) as “the tailoring of medical treatment to the individual characteristics of each patient to classify individuals into subpopulations that differ in their susceptibility to a particular disease or their response to a specific treatment [3]”. *Thus, products and diagnostics developed through Precision Medicine facilitate the practice of Personalized Medicine*.

## Precision medicine case examples

### Developing a therapeutic for a targeted population: Crizotinib

Oncology is the frontier of Precision Medicine and typically focus is on identification of *gene fusions* (e.g. crizotinib, an ALK inhibitor for ALK fusion proteins in lung cancer), *mutations* (as in the case of vemurafinib, a BRAF inhibitor for melanoma patients with the BRAF V600E mutation) and *protein over- expression* (e.g. Trastuzumab a HER2-targeted antibody for breast cancer). Crizotinib was identified in 2005 as a cMET (and ALK/ROS1) kinase inhibitor. In 2007, contemporaneously with dose escalation in Phase I studies, academic researchers in Japan found that about 5% of patients with non-small cell lung cancer (NSCLC), typically younger non-smokers, carry an EML4-ALK fusion gene leading to constitutive activation of ALK kinase. The Phase I protocol was amended to include patients with ALK-positive tumors, and within a few months of enrolling the first patients with the ALK mutation, objective tumor responses were seen in ALK-positive NSCLC patients. An impressive objective response rate (ORR) of over 60% and a disease control rate of over 80% which was significantly better than the expected ORR of less than 20% in matched, historical control patients were observed. Based on these data, a rolling submission in the first half of 2011 to the US FDA led to accelerated approval in less than 6 months following submission. The ability to respond quickly to the ALK rearrangement discovery in 2007, along with the successful development of a FDA-approved ALK companion diagnostic assay was instrumental in enabling FDA approval only five years after the start of the first clinical studies. Had the clinical trials been conducted in a non-selected NSCLC patient population, the strong clinical efficacy would likely not have been evident in Phase I/II studies, the therapeutic benefit likewise may not have been seen in a randomized Phase III study, and this compound might never have become a medicine. This success demonstrates the potential and value of Precision Medicine.

### Leveraging insight from human genetics to create novel therapeutics: PCSK9

The breakthrough in high throughput human genetics has enabled easy identification of the genetic cause for many “extreme diseases phenotypes”. This insight may lead to treatment of affected individuals as well as broader patient populations. One example of such an extreme phenotype relates to individuals with a rare loss-of-function mutation in the PCSK9 *gene* – but who are otherwise normal. These individuals have very low LDL-cholesterol as well as a markedly reduced incidence of cardiovascular disease. Based on these genetic findings and the biological knowledge that normal PCSK9 protein in the blood inhibits cellular uptake of LDL cholesterol several companies have developed antibodies that binds to and reduces the function of PCSK9 protein. Early clinical data suggest that treatment with one such antibody RN316 results in very significant and long lasting declines in LDL- cholesterol in the majority of patients with elevated LDL cholesterol. If these preliminary data are confirmed in broader testing, RN316 therapy may be particularly useful for treating high risk patients who do not respond adequately to intensive statin therapy.

### Leveraging information about cytokine signaling pathways to create novel anti-inflammatory drugs: Lebrikizumab and tofacitinib

Direct targeting of pro-inflammatory cytokines has delivered several therapeutics notably TNFα and IL-6 neutralizing antibodies. In the case of lebrikizumab, an anti- IL-13 antibody, the underlying biology (e.g., IL-13 stimulated bronchial proteins) was used to identify a biomarker (periostin) for identification of Phase II trial subjects who benefited the most from the agent. This may allow smaller phase III trials with better statistical outcome in the targeted population of patients with moderate to severe asthma. The oral JAK inhibitor Tofacitinib takes a novel approach, targeting the intracellular signaling pathways that operate as hubs in the inflammatory cytokine network. More specifically JAK3 mediates signaling through the common gamma chain of IL-2, IL-4, IL-7, IL-9, IL-15 and IL-21 cytokine receptor complexes, explaining the broad action of tofacitinib. Tofacitinib is being investigated as a targeted immune-modulator and disease-modifying therapy for Rheumatoid Arthritis (RA) and has the potential to become an important new treatment for RA patients. Phase 3 data showed that patients who received 5 mg or 10 mg tofacitinib have a statistically significant and clinically meaningful improvement in disease scores (ACR20, ACR50 and ACR70) vs. placebo. Tofacitinib is also being explored as a potential treatment for psoriasis, and inflammatory bowel disease. Precision Medicine may further help define the positioning of tofacitinib in these diseases as it was in RA trials where patients were selected who had moderate to severe disease as measured by disease activity score to identify a population with significant disease burden and risk of progressive joint injury.

### Understanding the molecular basis of adverse drug events to create safer therapeutics

Another aspect of Precision Medicine is the role safety plays in selecting patients for treatment to avoid drug-related adverse events. Analysis of the FDA Table of Pharmacogenomic Biomarkers in Drug Labels (96 drugs in total) demonstrated that 62% of the biomarkers are used to exclude patients or adjust dose based on safety-related concerns, and 31% are used for efficacy.[4,5]. The majority of safety biomarkers measure polymorphisms in genes encoding metabolizing enzymes and drug transporters with the objective of achieving adequate drug exposure. Other safety biomarkers focusing on idiosyncratic safety issues most often measure enzyme deficiencies and immune response (e.g. HLA genes). For Pharmacodynamic adverse drug events, cetuximab and panitumumab are monoclonal antibodies for colorectal cancer that target EGFR. Patients with activating mutations in the KRAS gene downstream of EGFR receive no therapeutic benefit from treatment. Therefore, in these patients, treatment skews the risk-benefit profile toward toxicity without benefit.

## Strategic application of precision medicine

Precision Medicine success is predicated on the appropriate selection of projects that benefit from its application, ideally fulfilling the following criteria:

· Strong human biology data package with evidence that the drug target is a critical driver of disease and its modulation is likely to produce a meaningful clinical benefit.

· Evidence of downstream pharmacodynamic effects as a consequence of target engagement.

· Biomarkers predictive of clinical benefit (both for efficacy and safety/toxicity) to allow population stratification and associated diagnostic markers if appropriate.

## Biomarker and diagnostic marker discovery and development

The development of biomarkers - some of which may ultimately translate into companion diagnostics - is a fundamental requirement to enable Precision Medicine. Broadly speaking, biomarkers can be categorized in terms of disease, mechanism or outcome. Those that are prognostic of disease progression or predictive of outcome will form the basis of companion diagnostics. It is critical to develop biomarkers at the earliest stages of drug discovery and, where based on preclinical models, translate them effectively into the clinic. The development of biomarkers to segment diseases into better understood molecularly-characterized subtypes depends on access to well-defined patient populations and patient biologic samples along with longitudinal clinical data and medical and treatment histories. Clinical trials or methodology studies will be required not only to demonstrate fit-for-purpose validation or qualification of specific biomarker candidates, but also to identify new biomarker candidates related to treatment outcomes, which in turn can be used for early research purposes. The development of a comprehensive biomarker plan at the very early stages (e.g. lead development) of drug development enables the generation of appropriate pre-clinical and translational markers and delivery of a qualified, fit-for-purpose biomarker to be incorporated into clinical trials.

Like therapies, biomarkers are validated in clinical trials. A typical biomarker is first evaluated in Phase I/II clinical trials. If the preliminary data indicates a clinically significant difference in outcome for patients based on a given biomarker, its predictive utility needs to be confirmed (replicated) in a prospectively designed study. A clinically significant improvement in outcome (therapeutic index) would be required for the biomarker to be validated for use in patient selection. Some of these biomarkers may be translated into a companion diagnostic that can be used to identify an appropriate subpopulation that should (or should not) receive treatment. Ideally, a drug and its companion diagnostic would be developed contemporaneously with the clinical relevance and technical performance criteria established using data from the clinical development program before NDA filing.

## Enabling technologies - creation of a strong data package

The ultimate end product of any R&D program is data that can support regulatory and intellectual property filings, inform a product label and support the manufacture and sale of the drug. Generation and advanced analysis of molecular and clinical data from studies are primary enablers for Precision Medicine decision making. Internal and external information can support and define decisions regarding target selection, pre-clinical and clinical experiment design, biomarker identification, disease modeling, and patient stratification. A wealth of human biology insight may be found in clinical, medical and basic biology data sets in academia and in pharmaceutical companies. These data are obtained from a range of resources including clinical trials, electronic medical records, personal health records, ‘omics’, sequencing and genotyping experiments. Opportunities exist to develop shared industry solutions relating to validation of markers, and to collaborate with commercial and not-for-profit entities on bio-specimens, clinical data, registries and other data relating to human biology.

Advances in lab technology over the last 10 years have increased the scale of internal and external data generation and increased the relevance to pharmaceutical companies. This trend will continue providing increasingly granular insight into the molecular drivers and correlates of clinical outcomes. Biomarker technologies that generate quantitative data, can be performed on small amounts of tissue (e.g. tumor biopsies), are multiplexable and/or allow for in vivo or minimally invasive assessment are particularly attractive.

## Regulatory considerations

Precision Medicine will hopefully lead to scientifically selected, well-defined populations enriched for responders, enabling smaller databases to support efficacy. The appropriate selection of patient populations for clinical trials has the potential to lead to earlier regulatory submissions, shorter and more predictable timing to approvals, and faster and more focused product launches. A higher rate of new drug approvals may also be expected due to stratification of populations when efficacy or risk considerations differ in various subpopulations, resulting in more compelling overall product risk/benefit (therapeutic index) versus current therapies, and better odds of approval. These benefits, however, come with some challenges. First, labels will be more narrowly crafted to focus the approved indication on the appropriate population . In addition, the regulatory pathway of novel therapies developed through Precision Medicine may be complicated by the accompanying selection method by which the population is defined. If a novel biomarker is to be used, and the product’s labeling defines the approved population by this measure, regulations require that the means of measuring the biomarker be commercially available. Increasingly, this is driving simultaneous drug and companion diagnostics development programs.

## Commercial and market access considerations

Precision Medicine requires careful evaluation of the commercial and market access landscape to accurately identify the commercial opportunity. Therapies developed through Precision Medicine may command a higher price and greater reimbursement, driven by enhanced value of therapy to patient and payer, improved patient compliance and increased level of trust and confidence among all stakeholders and reduced number of failures. In addition, there may be fewer adverse events and therefore less potential for market withdrawal and fewer label restrictions. However, these advantages are balanced by a more focused and fragmented market that might limit sales potential.

For these reasons, it is important to build a strong case for the medicine’s clinical benefit and therapeutic index in the defined patient population. Such a case could be supported through health economic considerations and/or health technology assessments which are increasingly being used by public health bodies to make recommendations on reimbursement of medicines and devices. From a payer perspective, there are many benefits of Precision Medicine that enhance the value proposition. These include better risk estimation and treatment optimization, diagnostics that can screen high risk patients to identify a specific disease early, and tests that provide healthcare providers and payers with a broad risk profile for a specific disease to identify appropriate patients for a given targeted therapy. Importantly, the ability to identify those patients who will have higher treatment response rates and will experience fewer adverse events and/or side effects will serve as a powerful driver of value as payers balance increasing healthcare costs against healthcare funding constraints and shrinking budgets. Payers are wary of covering diagnostics when there is a not a clear link to a health economics or treatment benefit, so the scientific and commercial rationale for its use must be sound.

## Legal and ethical considerations

Appropriate handling of the ethical and legal challenges for clinical trial investigators, regulatory authorities, patients, payers and other stakeholders that arise out of Precision Medicine is imperative to increase public confidence, the viability of data collection efforts and successful commercialization of personalized medicine products. A key area relates to privacy, confidentiality and patients’ rights issues, and the evolving ethical issues around collection of samples and data for future, “secondary” research that could involve new subpopulations, biomarkers, and other indications relevant to Precision Medicine. An additional consideration is the legal uncertainty regarding patentability and enforceability of personalized medicine method claims, which seek to cover the two-step process of determining the level of a biomarker (e.g., metabolite) in a diagnostic test and then administering the appropriate medication).
